# CRISPR/Cas12a combined with RPA for detection of *T. gondii* in mouse whole blood

**DOI:** 10.1186/s13071-023-05868-0

**Published:** 2023-07-30

**Authors:** Xiaofeng Wang, Miao Cheng, Shuqi Yang, Chen Xing, Qian Li, Yating Zhu, Yongsheng Ji, Yinan Du

**Affiliations:** 1 The Key Laboratory of Microbiology and Parasitology of Anhui Province, the Key Laboratory of Zoonoses of High Institutions in Anhui, Department of Pathogen Biology, School of Basic Medical Sciences, Anhui Medical University, Hefei, China; 2 School of Basic Medical Sciences, Division of Life Sciences and Medicine, University of Science and Technology of China, Hefei, China

**Keywords:** *T. gondii*, CRISPR/Cas12a, Nucleic acid testing, LFS, Mouse blood

## Abstract

**Background:**

*Toxoplasma gondii* is an opportunistic protozoan that is ubiquitous in humans and animals. It can invade any human organ and cause severe diseases, including toxoplasma ophthalmopathy, meningoencephalitis, and liver necrosis. Porcine toxoplasmosis is prevalent in China. CRISPR (Clustered Regularly Interspaced Short Palindromic Repeats) and Cas (CRISPR-Associated Protein) systems are widely used for gene editing and pathogen detection. CRISPR-based diagnostics are molecular assays that have been developed to detect parasites with high sensitivity and specificity.

**Methods:**

This study aimed to establish a combined CRISPR/Cas12a and RPA rapid detection method for *T. gondii* by targeting the *B1* gene and 529 bp repeat element (529 RE). The detection results could be visualized by the fluorescence or lateral flow strips (LFS). The sensitivity and specificity of the method were evaluated, and *T. gondii*-infected mouse blood was used for detection.

**Results:**

The results indicated that the established method for *T. gondii* detection was satisfactory, with a detection limit of 1.5 cp/μl for the two loci. Moreover, the *B1* gene could detect 1 tachyzoite per reaction, and the 529 RE could detect 0.1 tachyzoite per reaction, consistently with the highly sensitive nested polymerase chain reaction (PCR) results. The method was suitable for strains, including RH, and did not cross-react with other protozoa DNA with similar habits. The *T. gondii*-infected mouse blood samples were all positive for *T. gondii* at 1, 3, and 5 days post infection (dpi).

**Conclusions:**

This study established a rapid, sensitive, and time-saving DNA detection method for *T. gondii* that has the potential to be an alternative tool for *T. gondii* detection in the field.

**Graphical abstract:**

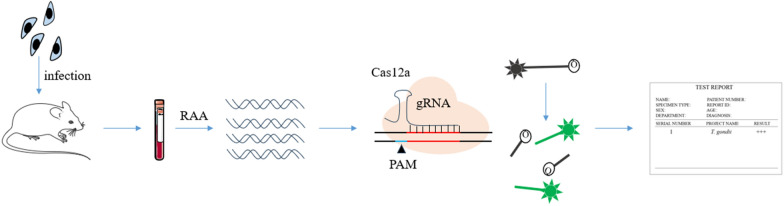

**Supplementary Information:**

The online version contains supplementary material available at 10.1186/s13071-023-05868-0.

## Introduction

Toxoplasmosis is a human-animal disease caused by a specialized intracellular parasite, *Toxoplasma gondii*, with a worldwide distribution [1]. *Toxoplasma gondii gondii* can infect all warm-blooded animals, including humans [2]. In immunocompetent individuals, infection with *T. gondii* can cause flu-like symptoms that persist for weeks to months before resolving. However, immunocompromised individuals, such as those with acquired immune deficiency syndrome (AIDS), the elderly, and those taking immunosuppressive drugs, are at a higher risk of severe illness and even death following *T. gondii* infection [3]. Pregnant women infected with *T. gondii* are also at risk of congenital toxoplasmosis in the fetus, which can lead to a range of adverse outcomes such as abortion, malformation, or stillbirth [4]. The serological positive rate of *T. gondii* in humans in some regions of China has been reported to be as high as 23.41%, indicating that the infection is widespread in the country [5]. In addition to its impact on human health, *T. gondii* has a significant impact on animal husbandry, with studies showing that the global seroprevalence of *T. gondii* in pigs is 19% [6]. Infection with *T. gondii* in pigs can exhibit a range of symptoms, including missed fever, diarrhea, pulmonary edema, and abortion in sows. Given that pork is a primary source of meat for humans, porcine toxoplasmosis increases the possibility of human *T. gondii* infection. Prevention and screening of *T. gondii* are becoming increasingly important. However, the lack of specific clinical symptoms of toxoplasmosis poses a challenge for diagnosis, highlighting the need for the development of more effective *T. gondii* detection methods.

*Toxoplasma gondii gondii* detection can be achieved through a variety of methods, including etiological, immunological, and molecular techniques. Etiological methods, such as peritoneal fluid smears and histological section staining, are time-consuming and have a high false-negative rate. Immunological tests, such as enzyme-linked immunosorbent assay (ELISA), are the most common methods for detecting toxoplasmosis. While ELISA is simple and rapid, patients in the window period of infection may be missedm and there are various interfering factors, such as rheumatoid factor [7]. Furthermore, patients with chronic lymphocytic leukemia (CLL) and secondary hypogammaglobulinemia may produce false-negative results due to their inability to produce IgG antibodies [8]. In molecular biology, PCR technology is the most widely used. Burg et al. successfully used PCR technology to detect *T. gondii* DNA from blood using *B1* gene as a target gene in the twentieth century [9]. The use of conserved genes, such as the *P30* gene and the *SAG1* gene, has been successful in detecting *T. gondii* [10, 11]. Quantitative PCR (Q-PCR) is a useful derivative of PCR that can be used for both qualitative and quantitative analysis. However, Q-PCR requires expensive and precise instruments, limiting its use to the laboratory, making it unsuitable for field applications.

In addition to the high specificity, high sensitivity, and short detection window, the ideal pathogen detection should also ensure the portability and low cost of detection equipment. In recent years, the emergence of isothermal amplification techniques such as loop-mediated isothermal amplification (LAMP) and recombinase polymerase amplification (RPA) [12, 13], as well as the discovery of Cas12a, Cas13a, and Cas14 in the CRISPR/Cas family [14–16], have provided viable approaches for point-of-care testing (POCT). RPA can achieve exponential amplification of template DNA in a short time and conventional temperature environment and eliminate the dependence on PCR instrument. The CRISPR/Cas system has shown potential in pathogen detection, with the Cas12a system able to non-specifically cleave other single-stranded DNA in the system after specific cleavage of double-stranded DNA by the crRNA targeting the target sequence [17]. In 2018, Doudna et al. established the Cas12a-based DNA endonuclease-targeted CRISPR trans-reporter system (DETECTR), which has been used to rapidly detect pathogens such as SARS-CoV-2 and African swine fever virus (ASFV) [14, 18, 19]. Moreover, CRISPR-based detection methods have been applied to the detection of *Cryptosporidium parvum* and *Plasmodium* [20–22], indicating the broad potential of CRISPR in parasite detection.

The *B1* gene of *T. gondii*, with 35 copies in the parasite's genomic DNA, is now considered the most used gene in the field of *T. gondii* nucleic acid detection [7, 23, 24]. Similarly, the 529 RE gene fragment, with a repetition rate ranging from 200 to 300 copies in the *T. gondii* genome, is thought to contribute to the improved sensitivity and specificity of the detection [25]. In this study, we have developed a molecular detection method for *T. gondii B1* gene and 529 RE using the newly developed DETECTR technology and compared its efficacy with that of the nested PCR approach. The established method was used to detect the blood samples of host animals. We demonstrate that our method is characterized by a high level of simplicity, speed, and accuracy and is for point-of-care testing (POCT), using of simple visualization techniques like placing the test tube in a dark box under UV light in an outdoor environment or application of a lateral flow strip (LFS) for direct naked-eye readout of results.

## Materials and methods

### Mice and parasites

The strains RH, Pru, wh3, and wh6, utilized in the present investigation, were generously provided by the Anhui Key Laboratory of Zoonoses, Anhui Medical University. The DNA of *C. parvum* and *Babesia* were procured from Shanghai Veterinary Research Institute, Chinese Academy of Agricultural Sciences. Furthermore, *Plasmodium* was obtained from Chao Zhang of the Department of Pathogen Biology, Anhui Medical University. Six- to eight-week-old female BALB/c mice were purchased from the Animal Experiment Center of Anhui Medical University.

### Protein expression and purification

Cas12a plasmid (pMBP-LbCas12a) was purchased from Addgene (Plasmid #113431). Expression vectors containing N-terminal 10 × His-tag, MBP, and TEV protease cleavage sites were transformed into receptor cells *Escherichia coli* BL21 (DE3). Cells were cultured in LB medium at 37 ℃ until logarithmic growth phase; 8 ml cells was added to 400 ml LB medium at 16 ℃, 200 rpm, and incubated overnight. Then cells were collected and centrifuged. The supernatant was discarded and resuspended in lysis buffer (50 mM Tris–HCl, 500 mM NaCl, 5% (v/v) glycerol, 1 mM TCEP, 0.5 mM PMSF, and 0.25 mg/ml lysozyme, pH 7.5) and lysed by ultrasonication. The precipitate was removed by centrifugation with 10,000 rcf for 1 h at 4 °C. The protein supernatant was filtered after centrifugation using a 0.22-μm bacterial filter. The filtrate was added to a Ni–NTA purification resin pre-loaded column (Sangon Biotech, Shanghai, China), linear elution with buffer. After holding overnight, TEV cleavage at 4 °C was carried out to remove the 10 × His-MBP tag, and the sample was loaded into a heparin column (GE, Hi-Trap) for further purification to obtain Cas12a protein. The buffer was replaced with the final storage solution for Cas12a (20 mM Tris–HCl, 2.0 M NaCl, pH 8.5) for protein concentration by Amicon® Ultra-4 centrifugal filtration device (Millipore, China). The BCA method was used for concentration determination, identification was made using sodium dodecyl sulfate polyacrylamide gel electrophoresis (SDS-PAGE) (Additional file 1: Fig. S1), with storage at − 20 °C.

### Design and preparation of crRNA

Conserved and specific *T. gondii* genome sequences were identified using the National Center of Biotechnology Information (NCBI), and the final selection was the *T. gondii B1* gene (GenBank: AF179871) and 529 RE (GenBank: AF146527) as the target genes. After 66 nt ssDNA oligonucleotide was synthesized by the company (Additional file 1: Table S1), ssDNA oligonucleotide was transcribed into ssRNA in vitro using HiScribeT7 Quick High Yield RNA Synthesis Kit (NEB, USA). The transcription system was 10 μl NTP buffer mix, 2 μl T7 RNA polymerase mix, and 1 ug ssDNA oligonucleotide, and 20 μl was reached with RNase-free water. Incubation was carried out at 37 ℃ for 7 h. After incubation, 2 μl DNaseI was added, and water was added to reach a volume of 50 μl. The DNA template was removed by incubation at 37 ℃ for 15 min. The resulting crRNA was obtained by RNA purification using Monarch® RNA Cleanup Kit (NEB, USA). Attention should be paid to prevent RNase contamination during all operations. The purified product was used for concentration determination and dispensed into a − 80 ℃ cryogenic refrigerator for storage before use.

### DNA extraction, recombinant plasmid constructs, and tachyzoite DNA dilution

The suspension containing the tachyzoites of *T. gondii* RH was centrifuged at 2000 rpm for 5 min, and the supernatant was discarded. *Toxoplasma gondii* DNA was extracted using the TIANamp Genomic DNA Kit (Tiangen, China). Target genes were amplified by PCR primers (Additional file 1: Table S2) and purified with a DNA purification kit (Axygen, China). The purification product was inserted into T-Vector pMD19 vector by T-A cloning. After transformation to the DH5α-receptive state, extraction was performed with the Axygen Plasmid Small Volume DNA Extraction Kit (Axygen, China). Concentration was measured by NanoDrop 2000 (Thermo Fisher, USA), recorded, and then stored at − 20 °C.

DNA was extracted after counting 10^6^ *T. gondii* tachyzoites with a cell counting plate, and genomic DNA was diluted doubling with ddH_2_O to contain 10^5^ tachyzoite DNA per tube to 10^–1^ tachyzoite DNA for subsequent testing.

### DETECTR assays

The RPA Nucleic Acid Amplification Kit (Hangzhou ZC, China) was used for the isothermal amplification of nucleic acids. The total reaction system for amplification was 50 μl; 41.5 μl Buffer A solution, 2 μl (10 μM) each forward and reverse primers (Additional file 1: Table S3), and 2 μl template DNA were added to an RPA unit tube containing enzyme lyophilized powder; 2.5 μl Buffer B solution was added to its cap. The cap was covered, inverted, and mixed 5–6 times. The reaction tube was bathed in 37 ℃ water for 30 min to obtain an amplification product that could be used for subsequent CRISPR assays.

The metal PCR tube rack required for CRISPR reactions was precooled in advance in a − 80 °C refrigerator, after which the following system was prepared on the cooled PCR tube rack: 1 μM LbCas12a, 2.5 μM crRNA, and 2 μl 10 × NEB buffer 2.1, and finally 20 μl was made up with RNase-free water. Ten minutes incubation at 37 °C was followed by the addition of 0.5 μM DNA fluorescent probe (5'-FAM-TTATTATT-Bio-3'). Taking 18 μl of the above reaction system, 2 μl of RPA amplification product was added to it, and the fluorescence was monitored at the FAM channel using BioRad CFX96 to read the fluorescence value.

### Lateral flow strips

Aspirated 2 μl of the completed pre-amplified sample was added to the CRISPR/Cas12a cleavage reaction system; 20 μl of the reaction system was incubated for 1 h at 37 °C. Then, 80 μl of 1 × PBS was added and mixed well; 100 μl was aspirated to the sample pad and the results observed after 5–10 min at room temperature.

### Nested PCR

The nested PCR primers (Additional file 1: Table S4) were used as reported in the article by Shirzad Fallahi et al. [26]. The length of the nested PCR product was 194 bp for the *B1* gene and 164 bp for the 529 RE. The first round of PCR reaction system was 50 µl, containing 10 µl 5 × PrimeSTAR buffer, 4 µl dNTP Mix, 1 µl (10 µM) each of upstream and downstream primers, 1 µl DNA template, 1 µl Taq DNA polymerase, and 50 µl made up with ddH_2_O.

The amplification procedure was as follows: initial denaturation was done at 95 °C for 5 min, followed by 34 cycles of denaturation (10 s at 98 °C), annealing (15 s at 56 °C), and extension (60 s/kb at 72 °C), at the end of the cycle extended at 72 °C for 5 min. The products of the first round were added to the system of the second round as the template of the second round, and the procedure was the same as in the first round. The products were visualized by agarose gel electrophoresis.

### Establishment of mouse model of *T. gondii* infection

Counting was performed using a cell counting plate. Each mouse was injected with 1000 tachyzoites, and the control group was injected with the same volume of saline. Five mice from each group were selected for eye blood sampling on 1, 3, and 5 dpi, and control mice were dissected and killed on the last day. The blood needed to be anticoagulated, after which 200 µl of anticoagulated blood was taken for testing, using samples from the saline group as a negative control. The use of animals was approved by the Experimental Animal Ethics Committee of Anhui Medical University.

## Results

### Design and optimization of DETECTR detection system for *T. gondii*

**Fig. 1:**
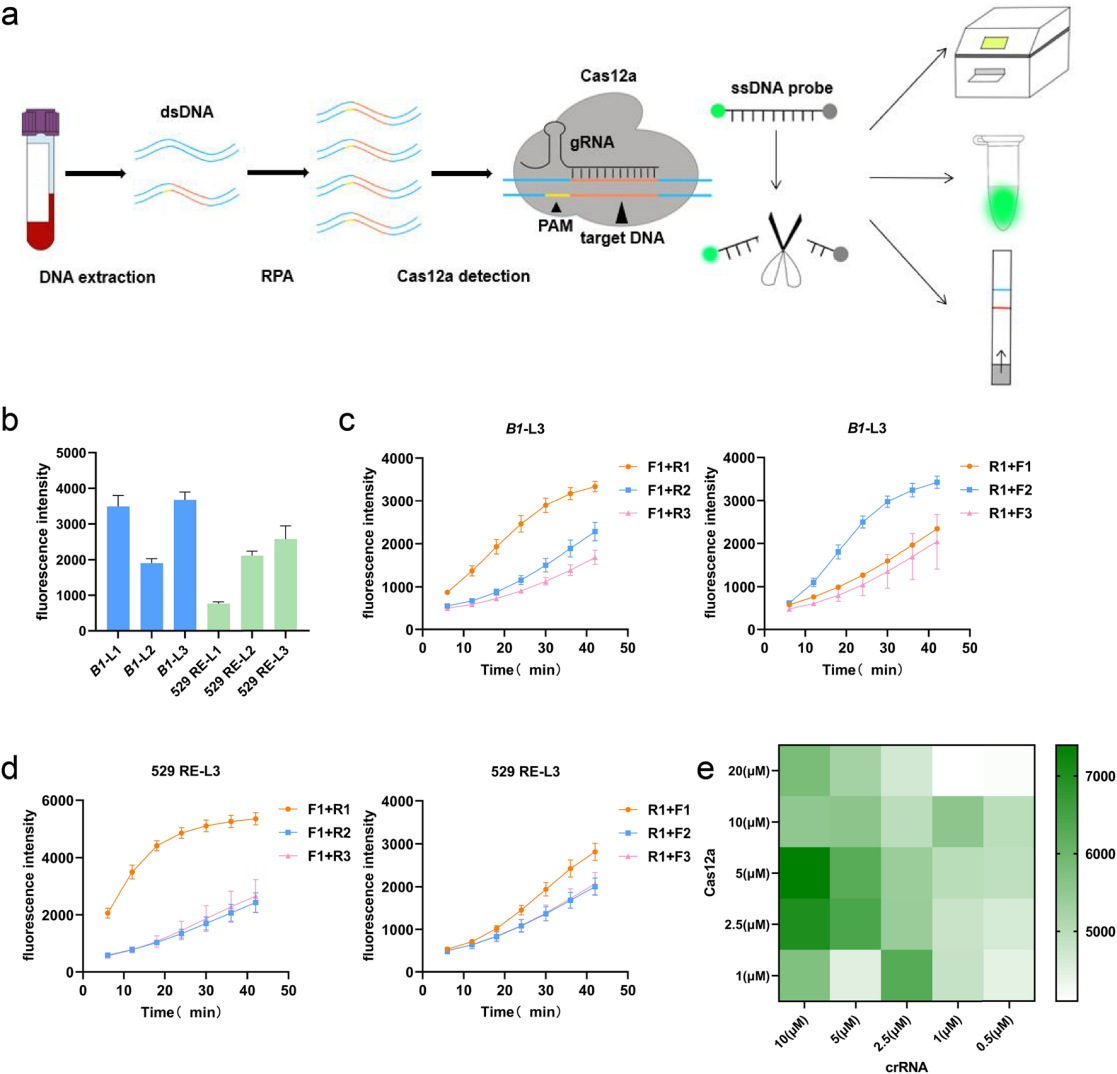
Establishment of CRISPR/Cas12a combined with RPA for detection of *Toxoplasma gondii*. **a** DETECTR workflow of *T. gondii*. **b** Comparison of different crRNAs targeting *B1*and 529 RE by fluorescence assay. **c** Screening of RPA primers for *B1*-L3. **d** Screening of RPA primers for 529 RE-L3. **e** Optimization of the concentration of Cas12a and crRNA

In this study, we developed a rapid detection method for *T. gondii* infection by combining RPA and CRISPR/Cas12a (Fig. 1a). The method involves three simple steps: nucleic acid extraction, RPA amplification, and CRISPR/Cas12a specific detection. To increase the sensitivity of the detection method for *T. gondii*, we conducted a comparison of targets, screening of primers, and system optimization. For the *B1* and 529 RE targets, we designed three crRNAs for each and evaluated their performance in the DETECTR system by assessing fluorescence intensity. Ultimately, we selected *B1*-L3 and 529 RE-L3 for detecting *T. gondii* (Fig. 1b). To identify the most efficient primers for RPA amplification, we designed three forward and reverse primers for each locus. We fixed the forward primers to screen out the optimal reverse primers and similarly screened out the forward primers (Fig. 1c, d). We optimized the system by adjusting the concentration of protein and crRNA and determined that the best detection effect was achieved when the protein final concentration was 5 μM and the crRNA final concentration was 10 μM. To reduce assay costs, we chose to use a Cas12a final concentration of 1 μM and a crRNA final concentration of 2.5 μM, which were employed in subsequent experiments (Fig. 1e).

### DETECTR has robust sensitivity for detecting *T. gondii*

**Fig. 2:**
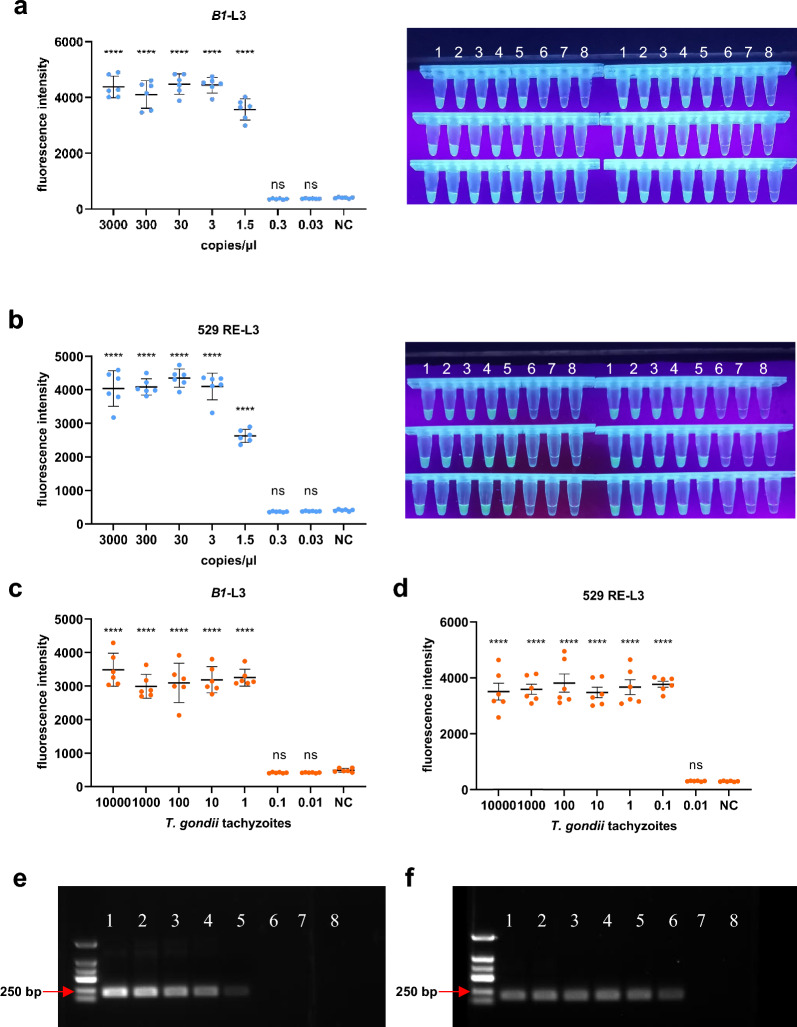
Sensitivity of the DETECTR system: (**a**) *B1*-DETECTR and (**b**) 529 RE-DETECTR reaction with plasmid standards. The tube shows the fluorescence signal of the sample under UV light. 1–8 represent 3000 cp/μl, 300 cp/μl, 30 cp/μl, 3 cp/μl, 1.5 cp/μl, 0.3 cp/μl, 0.03 cp/μl, negative control. **c** *B1*-DETECTR and **d**529 RE-DETECTR reaction with *T. gondii* tachyzoite DNA. **e** *B1*-nested PCR and **f** 529 RE-nested PCR reaction with *T. gondii* tachyzoite DNA. 1–7 represent 10,000, 1000, 100, 10, 1, 0.1, 0.01 tachyzoite DNA per reaction. ****P < 0.0001; ns, not significant

We evaluated the limit of detection of the DETECTR assay for *T. gondii* using recombinant plasmids and found that the detection sensitivity for the *B1* gene and 529 RE loci was as low as 1.5 cp/μl (Fig. 2a, b). Notably, positive samples emitted strong yellow-green fluorescence under UV, which was observable by the naked eye, making this method particularly suitable for use in resource-limited settings (Fig. 2a, b).

We further tested the DETECTR assay using serial dilutions of tachyzoite DNA and found that the detection limit was 1 tachyzoite per reaction for *B1* (Fig. 2c) and 0.1 tachyzoite per reaction for 529 RE (Fig. 2d); 529 RE was more sensitive than *B1*, perhaps because 529 RE has 200–300 copies in the *T. gondii* genome, while the *B1* gene has only 35 copies. Furthermore, the DETECTR assay was found to be comparable to nested PCR as both methods were able to detect 1 tachyzoite per reaction for *B1* (Fig. 2e), while 529 RE could detect 0.1 tachyzoite per reaction (Fig. 2f). These results suggested that the DETECTR assay was a sensitive and reliable method for the detection of *T. gondii* tachyzoite DNA, particularly when targeting the 529 RE locus.

### DETECTR has robust specificity for detecting *T. gondii*

**Fig. 3:**
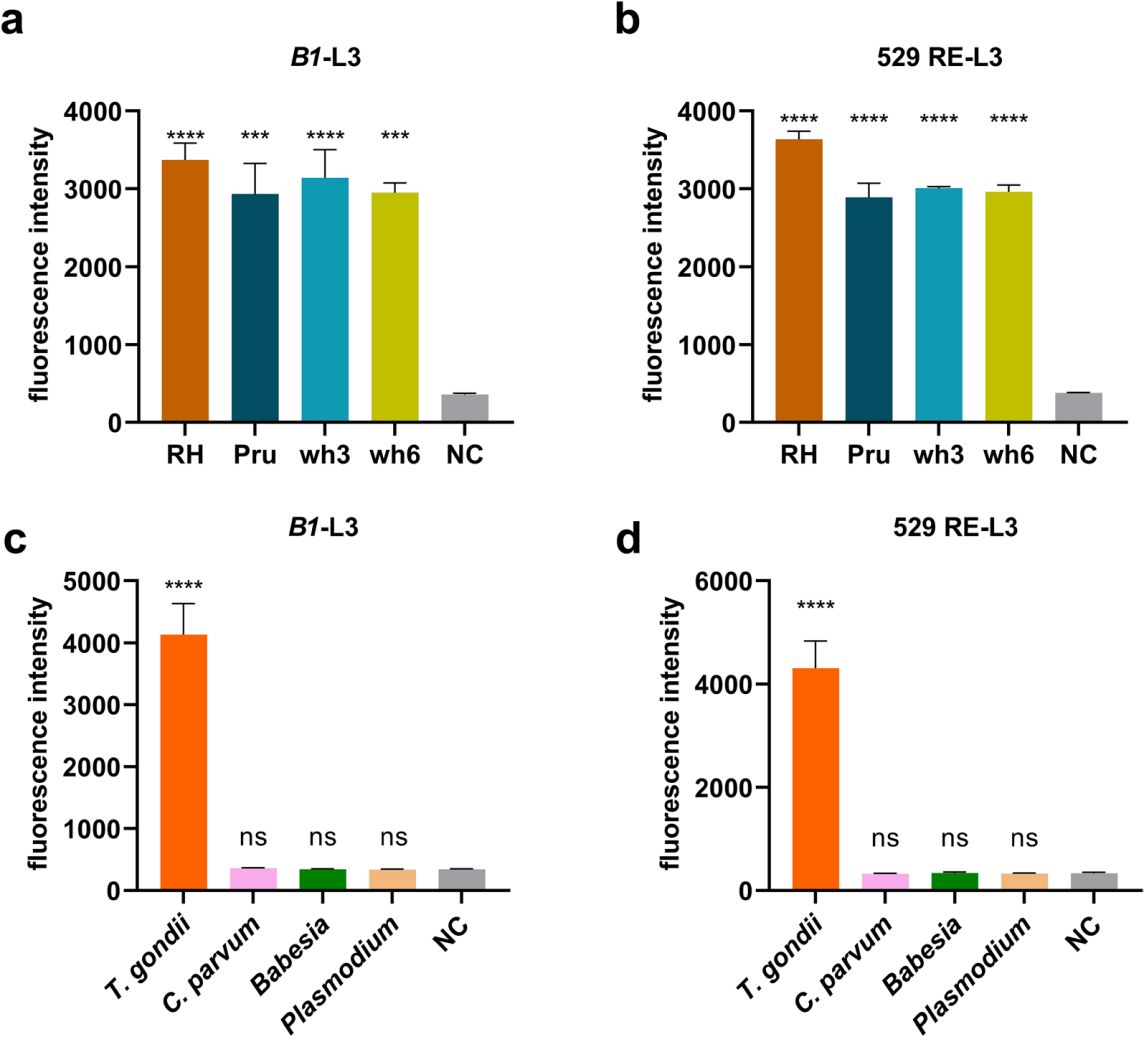
Specificity of the DETECTR system: (**a**) *B1*-DETECTR and (**b**)529 RE-DETECTR reaction of DNA of four different strains of *T. gondii*; all four strains can be detected. NC, negative control. **c** *B1*-DETECTR and **d**529 RE-DETECTR reaction of four types of parasite DNA, except DNA of *T. gondii*, could not be detected. NC, negative control; ***P < 0.001; ****P < 0.0001; ns, not significant

To investigate the universality of the DETECTR assay for *T. gondii*, we detected the DNA extracted from four different strains, including RH in type I, Pru in type II, and wh3 and wh6 strains of Chinese 1 genotype. The results revealed that this method was effective for all four strains, exhibiting strong fluorescence values, and there was no significant difference in the detection effect among the four strains (Fig. 3a, b).

In addition, we selected *Plasmodium*, *C. parvum*, and *Babesia*, which have similar habits or infection symptoms to *T. gondii*, for specific detection. The DETECTR system for *T. gondii* showed positivity only for *T. gondii* DNA, and the fluorescence values of the other three protozoa were consistent with those of the negative control group (Fig. 3c, d). This result was also evident under UV light (Additional file 1: Fig. S2). Hence, the DETECTR system demonstrated good specificity.

### DETECTR and nested PCR detection of mouse blood samples

**Fig. 4:**
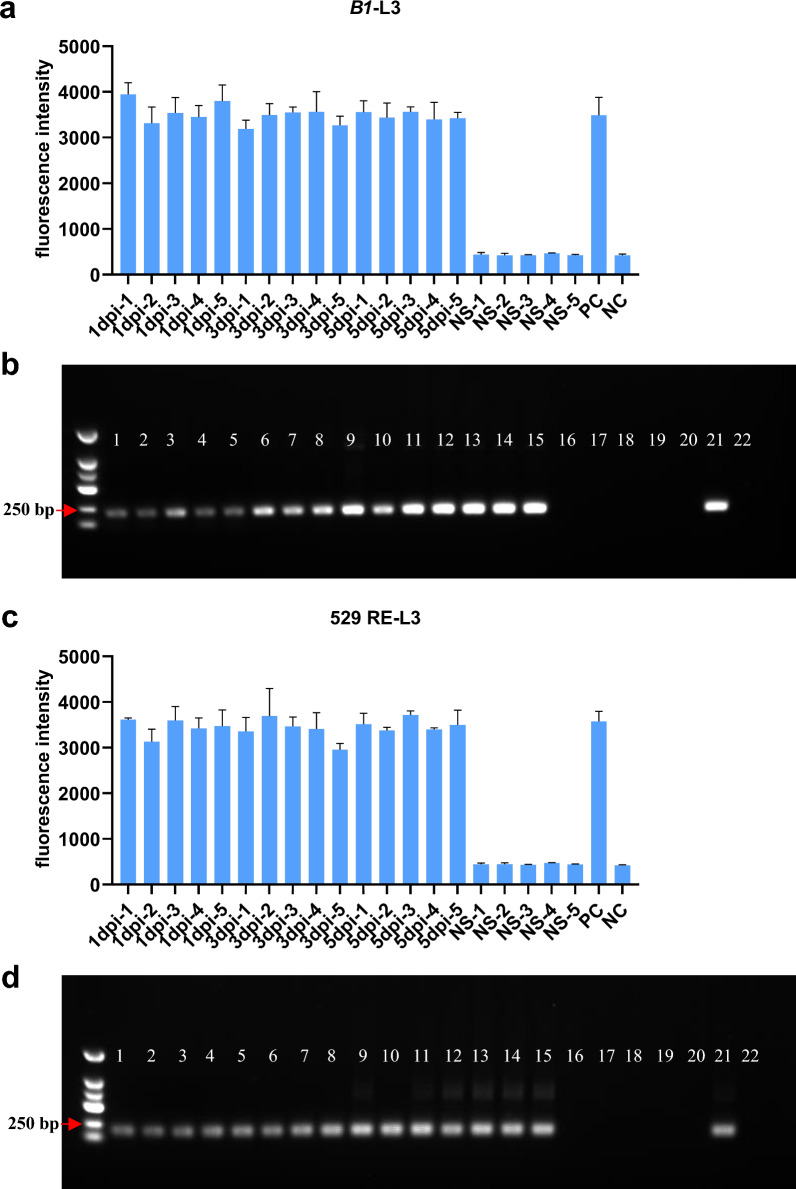
Analysis results of blood samples from *Toxoplasma gondii*-infected mice. **a** *B1*-DETECTR and **c** 529 RE-DETECTR reaction with mouse blood DNA. 1 dpi, 1 day post-infection mice #1; NS-1, normal saline #1; PC, positive control; NC, negative control; **b** *B1*-nested PCR and **d** 529 RE-nested PCR reaction with mouse blood DNA. 1–15 represent sample mouse blood samples collected 1, 3, 5 days post injection; 16–20 represent samples after saline injection; 21 represents positive control; 22 represents negative control

We have developed a murine model of toxoplasmosis by administering 1000 RH tachyzoites intraperitoneally to BALB/c mice. We employed the DETECTR system to perform rapid detection of *T. gondii* in whole blood samples collected from infected mice. Our findings demonstrated that *T. gondii* DNA could be successfully detected in all blood samples obtained on 1, 3, and 5 dpi, while the negative control group injected with saline produced negative results (Fig. 4a, c). Nested PCR was also performed on blood samples from mice infected with 1000 RH tachyzoites, and all samples were positive at 1, 3, and 5 dpi, while all samples in the control group were negative (Fig. 4b, d). Our study indicates that the DETECTR system is capable of detecting *T. gondii* infection in its early stages.

### Portability of DETECTR

**Fig. 5:**
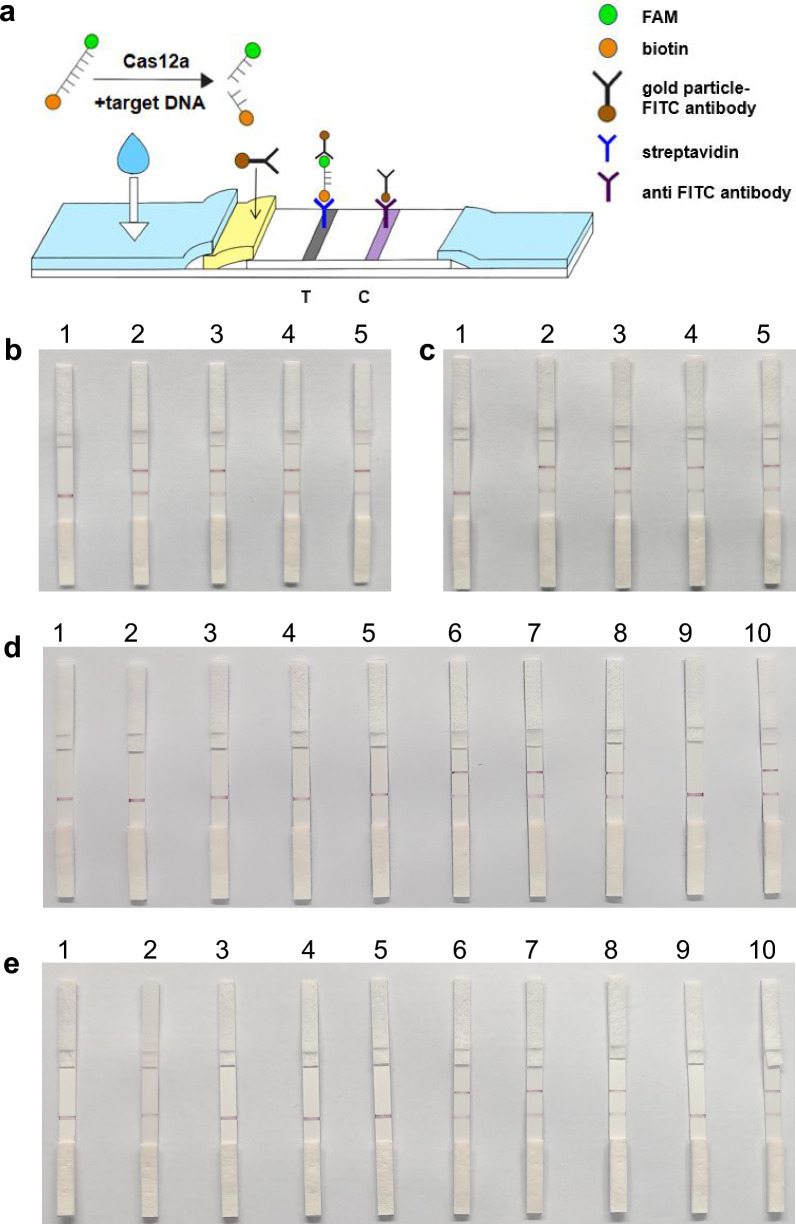
DETECTR-based LFS detection of *T. gondii*. **a** Principle of LFS. **b** *B1*-LFS and **c** 529 RE-LFS reaction with Specificity detection. 1–5 represent *Toxoplasma gondii*, *Cryptosporidium parvum*, *Babesia*, *Plasmodium*, negative control. **d** *B1*-LFS and **e** 529 RE-LFS reaction with mouse blood DNA. 1–5 represent 5 days post injection; 6–8 represent samples after saline injection; 9 represents positive control, 10 represents negative control

In this study, we combined the CRISPR/Cas12a-based DETECTR detection system with LFS to detect target DNA. The LFS technology [27] utilizes the principle of sandwich immunochromatography to detect the presence of target DNA in a sample (Fig. 5a). Specifically, the LFS incorporates a sample binding area, a test line, and a control line. Gold nanoparticle-coupled FITC antibodies are immobilized in the sample binding region, the test line is labeled with streptavidin, and the control line is labeled with anti-FITC antibodies. After adding the reaction system, the ssDNA reporter molecule (FITC-TTATTATT-biotin) can bind to the gold nanoparticle-coupled FITC antibody as a complex. If it is a negative sample, the complex is captured at the test line and both lines will show up; if it is a positive sample, Cas12a is activated and cleaves the reporter molecule, and the complex is not captured by the test line; only the control line will be shown. If neither or only the test line is shown, this indicates that the operation is incorrect or the test strip has failed, so the results are not informative. Overall, the developed method provides a promising tool for portable detection of target DNA in various settings.

In this study, we evaluated the sensitivity and specificity of LFS detection method for the diagnosis of *T. gondii* infection using the CRISPR-based detection system. Specifically, we mixed the amplified samples with a CRISPR detection system and incubated them for 1 h before performing the LFS. We then performed a specificity assay with the LFS and found that there was no cross-reactivity with other parasites, indicating the high specificity of the method (Fig. 5b, c). Moreover, we used the LFS to detect *T. gondii* in whole blood samples collected from five mice on the 5 dpi. Remarkably, all samples were positive for *T. gondii*, and the results were consistent with fluorescence detection (Fig. 5d, e). These findings collectively demonstrated that the LFS detection method exhibited good sensitivity and specificity for *T. gondii* detection and can be used as a promising tool for accurate diagnosis of *T. gondii* infection.

## Discussion

To date, there is no effective therapeutic drug available for *T. gondii* infection. Therefore, the development of a rapid and accurate detection method for this pathogen is crucial for disease control and prevention of its spread. In this study, we developed a novel *T. gondii* detection approach that combined RPA and CRISPR/Cas12a technology, utilizing fluorescence or LFS as the reporting method. We conducted a comparative analysis of the effectiveness of nested PCR and DETECTR assays targeting *B1* and 529 RE, respectively. Results indicated that DETECTR offered comparable sensitivity and specificity to nested PCR, but was more user-friendly because of the lack of complex technical requirements. The DETECTR reaction was performed at a consistent temperature of 37 °C, and the entire process did not necessitate complex technical expertise, facilitating prompt on-site detection of *T. gondii* DNA within a brief span of time.

At present, nucleic acid detection methods for pathogen identification primarily involve three main stages: sample processing, nucleic acid amplification, and signal transduction and reporting. Traditional amplification methods such as PCR and Q-PCR are time-consuming, taking up to 2 h or even longer, and require thermal cycling. Although LAMP is a constant temperature amplification method, its primer design is complex, and the amplification reaction requires incubation at 65 °C for 1 h [28]. In contrast, RPA primer design is simple, and the amplification reaction can be performed at 37 °C for only 20–30 min. Additionally, the CRISPR/Cas12a trans-cleavage system can be activated at 37 °C, and the entire reaction can be performed in a water bath, constant temperature incubator, or even in the palm of one's hand. The results can be read under UV light or through the use of lateral flow strips, making detection more feasible in non-laboratory settings.

In this investigation, *B1* and 529 RE were selected as targets due to their frequent use in nucleic acid detection of *T. gondii*. Upon recognition of the target double-stranded DNA, the CRISPR/Cas12a system was activated, leading to massive cleavage of the single-stranded DNA reporter, which served as a signal transducer for converting the target dsDNA recognition to the fluorescence or LFS bands [29, 30]. The combination of RPA amplification effect and DETECTR technology results in highly sensitive detection of *T. gondii* DNA.

The sensitivity of DETECTR assays for the detection of serially diluted recombinant plasmids can reach 1.5 cp/μl. For *T. gondii* tachyzoite DNA, DETECTR can detect a minimum of 1 tachyzoite by *B1* gene (35 copies) and 0.1 tachyzoite by 529 RE (200–300 copies), with 529 RE being a better choice than *B1*, as corroborated by Shirzad Fallahi and colleagues [26]. Notably, the high sensitivity of nested PCR [31] for *T. gondii* DNA detection is comparable to that of DETECTR, indicating that the DETECTR technology can identify low concentrations of *T. gondii* in the environment. Furthermore, when evaluating whole blood samples from mice, DETECTR could detect positive samples on 1 dpi and distinguish infected from uninfected samples. The method we have developed is mainly for the detection of host blood samples; the collection of blood samples is a minimally invasive or even non-invasive process, unlike tissue samples, which can cause harm to the host. This method has the potential to be an early and powerful diagnostic tool for *T. gondii* DNA.

The validation of the DETECTR assay's specificity for *T. gondii* detection was performed by analyzing the DNA of *Plasmodium*, *C. parvum*, and *Babesia*, which are parasites that are either similar in development to *T. gondii* (*C. parvum*) or can be present in blood samples (*Plasmodium*, *Babesia*). Notably, all results were negative for these parasites, with the exception of *T. gondii*, confirming the high specificity of the DETECTR assay for *T. gondii* detection.

In summary, DETECTR is a straightforward and speedy method for detecting *T. gondii* that can be applied to various settings with limited resources. In addition to the detection of infected animals, the detection of *T. gondii* DNA in the environment can also be performed, which has great potential as an alternative to current detection methods. In addition to the above advantages, DETECTR has some limitations in the DNA extraction stage. CRISPR-based nucleic acid detection systems have been demonstrated to be compatible with methods that rapidly expose nucleic acids, such as heating or chemical lysis [32]. Therefore, the subsequent research has focused on developing techniques to quickly release *T. gondii* DNA from samples without affecting detection sensitivity, thus further shortening the detection time. Because differential genes exist between *T. gondii* strains [33], leading to variations in virulence and pathogenicity, current typing methods rely on sequencing and some PCR-derived techniques [34, 35]. Strain-specific sgRNAs can be designed for CRISPR targeting, which can be particularly beneficial for identifying and typing *T. gondii* genes. In addition to its usefulness in detecting *T. gondii*, DETECTR technology holds great promise for broader applications in *T. gondii* research.

## Supplementary Information

**Additional file 1: Figure S1.** Protein expression and purification. Coomassie blue-stained acrylamide gel. **Figure S2.** Specificity of the DETECTR reaction. The left tube represents *B1*-L3; the right tube represents 529 RE-L3. **Table S1.** ssDNA oligonucleotide sequences. **Table S2.** PCR primers for the construction of standards. **Table S3.** RPA primers. **Table S4.** Nested PCR primers

## Data Availability

The original contributions presented in the study are included in the article/Supplementary material; further inquiries can be directed to the author, Xiaofeng Wang: wangxiaofeng9516@163.com.

## Abbreviations

CRISPR: Clustered Regularly Interspaced Short Palindromic Repeats
Cas: CRISPR-Associated Protein
529 RE: 529 bp repeat element
PCR: Polymerase chain reaction
AIDS: Acquired immune deficiency syndrome
ELISA: Enzyme-linked immunosorbent assay
CLL: Chronic lymphocytic leukemia
Q-PCR: Quantitative PCR
LAMP: Loop-mediated isothermal amplification
RPA: Recombinase polymerase amplification
DETECTR: DNA endonuclease-targeted CRISPR trans reporter
POCT: Point-of-care testing
NCBI: National Center of Biotechnology Information
LFS: Lateral flow strip
dpi: Day post infection
