# Supplementary material for: CRISPR/Cas12a combined with RPA for detection of T. gondii in mouse whole blood
Source: Parasit Vectors. 2023 Jul 30;16:256. doi: 10.1186/s13071-023-05868-0 (PMC10387196; doi:10.1186/s13071-023-05868-0)
Supplement: Supplementary file 1 — Additional file 1: Figure S1. Protein expression and purification. Coomassie blue-stained acrylamide gel. Figure S2. Specificity of the DETECTR reaction. The left tube represents B1-L3; the right tube represents 529 RE-L3. Table S1. ssDNA oligonucleotide sequences. Table S2. PCR primers for the construction of standards. Table S3. RPA primers. Table S4. Nested PCR primers [file 13071_2023_5868_MOESM1_ESM.pdf]

Additional file 1:

Figure S1.

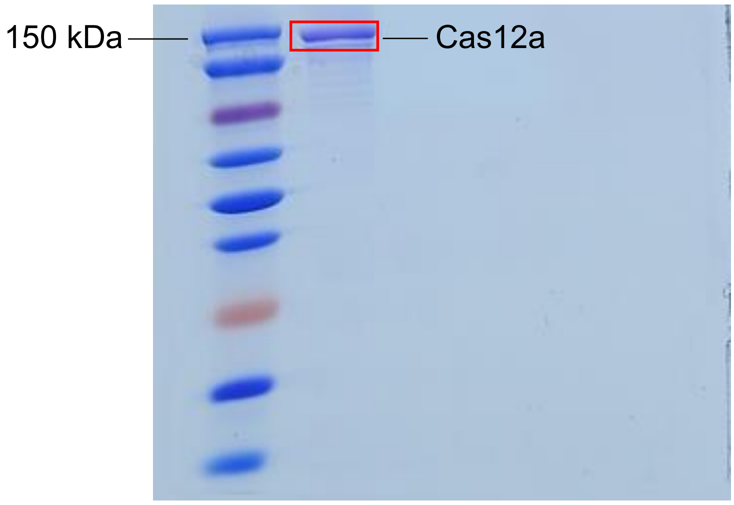

Figure S2.

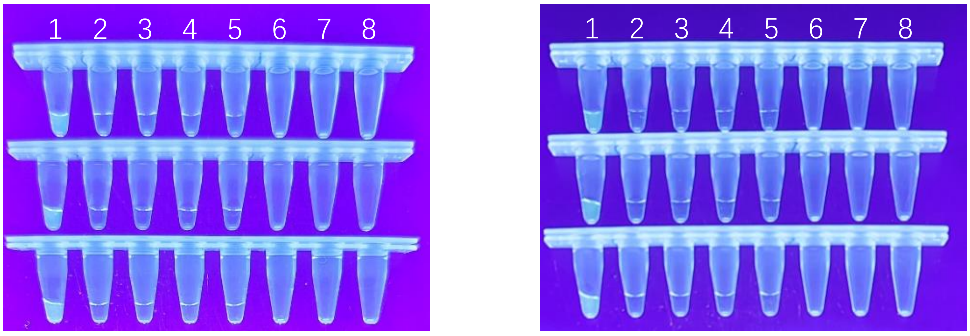

Table S1. ssDNA oligonucleotide sequences

ssDNA	5'-3'
B1-crRNA-L1	TCCAACTCCTGGTGTACTGCATCTACACTTAGTAGAA ATTACCCTATAGTGAGTCGTATTAATTTC
B1-crRNA-L2	ATATGTTCCCTCCTCTTCGCATCTACACTTAGTAGAAA TTACCCTATAGTGAGTCGTATTAATTTC

B1-crRNA-L3	GGTTGCCTCGAGTTCCTTCTATCTACACTTAGTAGAAATTACCCTATAGTGAGTCGTATTAATTTC
529 RE- crRNA-L1	GCACAGGCGAGCTCGCCTGTATCTACACTTAGTAGAAATTACCCTATAGTGAGTCGTATTAATTTC
529 RE- crRNA-L2	GCTGCTTTTCCTGGAGGGTGATCTACACTTAGTAGAAATTACCCTATAGTGAGTCGTATTAATTTC
529 RE- crRNA-L3	CTCTCCGCCATCACCACGAGATCTACACTTAGTAGAAATTACCCTATAGTGAGTCGTATTAATTTC
T7-3G IVT primer	GAAATTAATACGACTCACTATAGGG

Table S2. PCR primers for the construction of standards

Name	5'-3'
B1 -up-F	TCCTATCGCAACGGAGTTCT
B1-up-R	TTGGTGGTTCACTCCATCTC
B1 -down-F	CCGTTGATTCGTCTGATGGT
B1-down-R	ACGAAAGGAGAATGAGCGCA
529 RE -F	GCAGGGAGGAAGACGAAAGT
529 RE -R	GCAGACACAGTGCATCTGGA

Table S3. RPA amplification primers

Name	5'-3'
B1-L1-RPA-F1	TAGTATCGTGCGGCAATGTGCCACCTCG
B1-L1-RPA-F2	CAATGTGCCACCTCGCCTCTTGGGAGAA
B1-L1-RPA-F3	GAAGAGACGCTGCCGCTGTTTTGCAAATGA

B1-L1-RPA-R1	TCTACTCGACAATACGCTGCTTGAAGAG
B1-L1-RPA-R2	CCTTTCTGATCTACTCGACAATACGCTGC
B1-L1-RPA-R3	CGGATGCAGTTCCTTTCTGATCTACTCGACAA
B1-L2-RPA-F1	CAAGAAAATGAGATGCCTAGAGGAGACACAGC
B1-L2-RPA-F2	TGAGATGCCTAGAGGAGACACAGCGTGTTA
B1-L2-RPA-F3	GATGCCTAGAGGAGACACAGCGTGTTATGAAC
B1-L2-RPA-R1	TTAGCGACATGCGGCACGTCTCTTGTTCTT
B1-L2-RPA-R2	CTGAGCATCCCTTCCGATGGCTTAGCGACATG
B1-L2-RPA-R3	CTGTGCCATTTTCTGAGCATCCCTTCCGAT
B1-L3-RPA-F1	AAGTTCCGGTCGAGAGGCTAAACCACAA
B1-L3-RPA-F2	AGTGCAAACCATGCGCAGCCATCAGCTTA
B1-L3-RPA-F3	GCAGCCATCAGCTTAACAAAAGCAGTTG
B1-L3-RPA-R1	GCATGATTCTGCGTGGTGGGCTCGTTGAT
B1-L3-RPA-R2	TCCGCATTGTGCGGCTACTTTAGAAACGCT
B1-L3-RPA-R3	CCTTAGCATTCCGCATTGTGCGGCTACTTTAGAA
529 RE-L1-RPA-F1	TTTTTGACTCCGGCCCAGCTGCCTCTGT
529 RE-L1-RPA-F2	GGCCCAGCTGCCTCTGTCGGGATGAGAC
529 RE-L1-RPA-F3	AGACCGCGGAGCCGAAGTGCGTTTTCTTT
529 RE-L1-RPA-R1	CCCTTCGACTTCTGTCCCTTCTGTGGCT
529 RE-L1-RPA-R2	TGATATCTCTCCTCCAAGACGGCTGGAGGA
529 RE-L1-RPA-R3	TACAGTCCTGATATCTCTCCTCCAAGACGGCT
529 RE-L2-RPA-F1	CTGTAGATGAAGGCGAGGGTGAGGATGA
529 RE-L2-RPA-F2	AAGCGACGAGAGTCGGAGAGGGAGAAGA
529 RE-L2-RPA-F3	GAGAGTCGGAGAGGGAGAAGATGTTTCC
529 RE-L2-RPA-R1	TCGTCTCGTCTAGATCGCATTCCGGTGT
529 RE-L2-RPA-R2	ATTCTCTCCGCCATCACCACGAGGAAAGCGT
529 RE-L2-RPA-R3	TCTTCAATTCTCTCCGCCATCACCACGA
529 RE-L3-RPA-F1	GGAGAAGATGTTTCCGGCTTGGCTGCTT

529 RE-L3-RPA-F2	AGATGTTTCCGGCTTGGCTGCTTTTCCTGGA
529 RE-L3-RPA-F3	GAGACACCGGAATGCGATCCAGACGAGA
529 RE-L3-RPA-R1	TGTCTCCCTCGCCCTCTTCTCCACTCTT
529 RE-L3-RPA-R2	TCCCTTCGTCCAAGCCTCCGACTCTGTCT
529 RE-L3-RPA-R3	TCTCCTACCCCTCCTCCTCCCTTCGTCCAA

Table S4. Nested PCR primers

Name	5’-3’
B1-nested-F1	TCAAGCAGCGTATTGTCGAG
B1-nested-R1	CCGCAGCGACTTCTATCTCT
B1-nested-F2	GGAACTGCATCCGTTCATGA
B1-nested-R2	TCTTTAAAGCGTTCGTGGTC
529 RE-nested-F1	TCTTTAAAGCGTTCGTGGTC
529 RE-nested-R1	CTCCTCCCTTCGTCCAAGCCTCC
529 RE-nested-F2	AGGGACAGAAGTCGAAGGGG
529 RE-nested-R2	GCAGCCAAGCCGGAAACATC
